# Encapsulation of Electrically Conductive Apparel Fabrics: Effects on Performance

**DOI:** 10.3390/s20154243

**Published:** 2020-07-30

**Authors:** Sophie Wilson, Raechel Laing, Eng Wui Tan, Cheryl Wilson

**Affiliations:** 1 Materials Science and Technology, University of Otago, Dunedin 9016, New Zealand; raechel.laing@otago.ac.nz (R.L.); cheryl.wilson@otago.ac.nz (C.W.); 2 Department of Chemistry, University of Otago, Dunedin 9016, New Zealand; ewtan@chemistry.otago.ac.nz

**Keywords:** electrical conductivity, encapsulation, knit fabric, wool, cotton, wearable technology, grapheme

## Abstract

Electrically conductive fabrics are achieved by functionalizing with treatments such as graphene; however, these change conventional fabric properties and the treatments are typically not durable. Encapsulation may provide a solution for this, and the present work aims to address these challenges. Next-to-skin wool and cotton knit fabrics functionalized using graphene ink were encapsulated with three poly(dimethylsiloxane)-based products. Properties known to be critical in a next-to-skin application were investigated (fabric structure, moisture transfer, electrical conductivity, exposure to transient ambient conditions, wash, abrasion, and storage). Wool and cotton fabrics performed similarly. Electrical conductivity was conferred with the graphene treatment but decreased with encapsulation. Wetting and high humidity/low temperature resulted in an increase in electrical conductivity, while decreases in electrical conductivity were evident with wash, abrasion, and storage. Each encapsulant mitigated effects of exposures but these effects differed slightly. Moisture transfer changed with graphene and encapsulants. As key performance properties of the wool and cotton fabrics following treatment with graphene and an encapsulant differed from their initial state, use as a patch integrated as part of an upper body apparel item would be acceptable.

## 1. Introduction

Sensors produced from fabrics functionalized with electrical conductivity which respond when exposed to selected agents are of increasing interest. During use, apparel fabrics are typically exposed to varied transient conditions of the environment (temperature, humidity, abrasion) and for care (i.e., cleaning, storing) that may negatively affect and/or interfere with sensor performance: This was highlighted in a 2019 review [1]. Encapsulation can offer some protection to these functionalized fabrics whether they be self-contained devices, embroidered or woven/knit wires, or electrically conductive polymer/carbon treatments. Protection is achieved by creating a barrier between the surface of the material/device and the external environment, which is typically repellent to water and other agents. However, permeability of the fabrics needs to be maintained if the fabrics are to be used next to the skin in order to facilitate human thermoregulation [2,3].

Development of conductive fabrics using various forms of graphene has attracted high levels of research interest during the period ~2005 to 2020. Where the intended end application of these fabrics is apparel, many performance properties in addition to electrical conductivity need to be maintained or improved in order to be successful (e.g., resistant to abrasion, able to be stored with little change over time, able to withstand cleaning, resistant to dimensional change, permeable to air and to water vapor, retain some absorptive capability). While conductivity has been reported widely, there is little evidence on these critical apparel-related requirements: This paper addresses the gap.

A number of papers include treating fabrics with graphene (reduced graphene oxide [4,5,6,7,8,9,10,11,12,13,14,15,16,17,18,19,20,21,22], graphene nanoplatelets [23,24,25,26,27,28], nanoribbon [29], multilayer graphene [30], graphene [18,31,32,33]). These report electrical resistance, surface characteristics (SEM, TEM), and surface chemistry (FTIR, Raman) for example. Conferring electrical conductivity has been successful and performance as a sensor (e.g., to strain) has been demonstrated; however, challenges related to stability and durability for the end use typically remain. Change with wash [15,16,19,22,23,24,28,34,35,36,37,38,39,40,41,42,43] and with abrasion [7,16,34,35,43,44] have received some attention, but change with storage has not been well identified. There is little clarity and consistency in how and the extent to which properties critical to most wear-related applications change: Permeability to air [16,31,45,46,47], permeability to water vapor [16,46,47], moisture regain [46,48], thermal resistance [24,31,49], contact angle [8,15,28,31,33,50,51,52,53]. 

What we do know is that change does occur over time and with use, and that some form of encapsulation of the functionalized fabric has the potential to ameliorate these negative changes (e.g., encapsulated graphene-treated fabrics [25,26,30,54,55]). Responses to cleaning, abrasion, and storage of graphene-functionalized and -encapsulated fabrics have not been demonstrated. Comprehensive testing on the typical range of apparel fabric properties (i.e., fabric structure, moisture transfer, permeability, durability to cleaning, abrasion, storage) required for wearable applications has not been carried out. Additionally, in relation to electrical conductivity, quantifying effects of external factors which may affect sensor performance (i.e., fluctuations in temperature and humidity, presence of moisture) have not been identified. The present work aims to address these gaps using two single jersey apparel fabrics typical of next-to-skin applications treated with graphene ink and encapsulated.

Electrical properties of functionalized fabrics can be presented as one of two different forms: Electrical resistance/resistivity and/or conductance/conductivity. In the case of fabrics, reporting resistance relative to dimensions is preferable due to diverse specimen sizes which are functionalized. Ohm/square [27,28,56,57,58,59,60,61] or ohm/meter [30,57,61,62,63,64,65] are common in the scientific literature, the latter sometimes converted to Siemens/meter [22,66,67,68,69]. The Van de Pauw method [70] has been used to measure specific surface resistivity of fabrics mostly based on four-point probe measurements, especially when irregular material shapes are examined [56,71]. This method assumes specific conditions (i.e., homogenous, uniform thickness, isotropic, surface singly connected/no isolated holes), perhaps not met with a regularly shaped, non-uniform fabric structure of intertwined/interlaced yarns. Additionally, ensuring the area of electrode contact is, at minimum, an order of magnitude less than the specimen size can be difficult with use of small fabric specimens [57]. Resistivity measured by way of also measuring fabric dimensions (i.e., width, length, height) may therefore be most suited to the non-uniform structural properties [57]. In terms of comparability to related work, this method is also desirable. 

The two-probe method permits measurement of the electrical current passing through both the electrically conductive treatment and yarns/fibers present which remain partially non-treated/insulative, while the four-probe method/surface resistivity may primarily measure the current flowing through the conductive surface coating only. Measuring along the wales and courses of specimens with a large number of fine yarns per 10 mm and little variability in thickness and treatments which partially or completely fill interstitial spaces provides greater homogeneity compared to the not-treated fabric.

Conventional electronics which are not fastened to a fabric can be encapsulated for protection. Encapsulation processes used in the electronics industry (e.g., transfer molding, glob top encapsulation, and hot melt application [72]) exhibit some properties incompatible with textiles (e.g., high temperature and high pressure during processing). Therefore, identification and/or development of new processes for encapsulation of functionalized textiles is required. Commercial production and use is assumed the ultimate goal; thus, identification of a simple, effective method which maintains adequate performance of the fabric (i.e., minimal change to electrical conductivity, fabric properties, and durability) is desirable [73].

Different materials and processes for encapsulating functionalized fabrics have been identified: Immersion in polymers (e.g., poly(dimethylsiloxane) [54,73,74,75], silicon [76], epoxy resin [72,77], and Ecoflex™ [25,78]); application of tape (e.g., BEMIS™ Seam Tape, Bondex^®^/MD Mend and Repair™ Fabric Mending Tape [79]); and iron-on adhesives (e.g., Heat n Bond^®^ Iron-on adhesive [79]). Other commercially available encapsulation products designed for application by consumers, typically for water repellence/proofing, include Granger’s^®^ Clothing Repel, Scotchguard™ Fabric and Upholstery Protector, and 303^®^ Fabric Guard.

Double-sided encapsulation seems to provide protection to all surfaces, but can hinder heat and moisture transfer, which are important performance properties. Handle (i.e., how a fabric feels to the touch) may also be adversely affected. Single-sided encapsulation may be more desirable in maintaining handle for next-to-skin fabric applications. Exposure to heat and perspiration from human skin, and movement of the body continue to be factors likely to affect overall maintenance of electrically conductive fabrics.

Penetration of encapsulants beyond the fabric surface seems to reduce electrical conductivity [25,73,78] and change thermal properties [73]. A surface layer creates a physical insulative barrier between the fabric and connectors of electrical measurement devices (e.g., multimeter), therefore, edges of fabrics may need to remain non-encapsulated to fasten clips of such devices. The structure of the underlying fabric can affect encapsulant deposition within or on the fabric. For instance, poly(dimethylsiloxane) was evident in the yarn cross sections of knits but not evident in that of wovens [73]. Other fabric parameters such as stitch density, thickness, and mass per unit area were not controlled in this investigation but may differ among the fabrics and also affect reported outcomes. 

Encapsulant properties themselves reportedly affect patterns of deposition. Low viscosity encapsulants can fix to fabric, yarn, and fiber contours, filling interstitial spaces or increasing the effective diameter of the yarn, while high viscosity encapsulants tend to remain on the surface [73]. By changing the thickness of an encapsulating treatment, electrical conductivity and sensor performance can be also be altered [25,78]. Curing parameters can be manipulated to change deposition. Greater penetration into a fabric reportedly results from longer curing time with a lower temperature (e.g., “room temperature”, for 24 h), whereas treatments can be kept to the fabric surface with a shorter curing time and a higher temperature (e.g., 10 min at 100 °C) [73]. Investigating parameters to optimize performance is desirable, although application of some products is pre-determined by the product manufacturer.

Acceptability for use in next-to-skin apparel may be reduced with encapsulation due to changes in air and fiber ratios resulting in measurable and perceptible changes in permeability to water vapor and air. A small section or patch on a garment with this altered set of properties may be acceptable. Additionally, if encapsulation creates an impermeable layer, trapped moisture, heat, and vapors may adversely affect electrical conductivity. For example, Ecoflex^®^ encapsulated graphene treated cotton and wool weft knits exhibited similar heat transfer properties to one another in performance as wearable heaters [25]. Heat transfer with the elastomer was considered the determining factor [25]. Thus, the encapsulant dominated transfer properties. There may also be an associated lag time that would otherwise not be present [74], which is problematic if an immediate response as a sensor is sought.

Durability to wash has been reported in at least five published studies [72,73,77,79,80]. Ubiquitous for apparel, determining effects of wash is critical for items which require multiple uses and cleaning cycles. Satisfactory performance with wash has rarely if ever, been achieved: Using just a few repeat wash cycles does not simulate requirements for long term use. Cleaning may also be required following exposure as a sensor (i.e., perspiration, chemicals). Maintenance of performance (i.e., electrical conductivity) and leaching of products (graphene ink, encapsulants) and fibrous material in the environment are also critical when considering durability to wash.

Encapsulation products as additives to cleaning processes (e.g., Granger’s^®^ Performance Wash) are typical of available consumer products. Additional costs, treatments, and special wash cycles are inconvenient for normal wash procedures. Water repellence/proofing can reduce the number of washes an item requires over time, thus enhancing convenience for the end user and minimizing undesirable environmental effects related to wash cycles (e.g., fiber loss, treatment/chemical release [81,82]).

Extension and recovery cycles are a further measure of durability required for performance of some sensor applications, such as strain for movement, respiration, and heart rate [19,26,43,78,83,84,85,86,87,88,89,90,91,92,93,94,95,96,97,98], and are important predominantly for end-uses where there is large or frequent movement (joints, neck/garment openings). Other body locations, such as sensor positions on the upper part of the chest, are relatively stable and therefore extension/recovery is less relevant. Positioning at the upper chest section of an upper body garment is an anticipated application for fabric resulting from the present work, a site requiring minimal or no extensibility and recovery.

This paper focusses on encapsulation of next-to-skin fabrics (100% wool, 100% cotton, single jersey) previously functionalized with graphene ink. Encapsulation with polymers such as poly(dimethylsiloxane) warranted investigation because previously published work had indicated provision of protection with minimal adverse effects on fabric performance. Products suited to simple, effective application were considered desirable. Changes in performance were determined in an attempt to identify acceptability of encapsulants to protect functionalized fabrics designed to be worn next to the skin.

## 2. Materials and Methods

### 2.1. Materials and Treatments

Finished next-to-skin 100% merino wool single jersey (18.5 to 19.0 µm constructed in 2/72 Nm yarns with a Hercosett^®^ treatment) and 100% cotton single jersey, non-bleached and non-dyed were selected. Mass and thickness of the wool and cotton fabrics were 156 g/m^2^, 0.82 mm; 165 g/m^2^, 0.81 mm, respectively. Stitch density for wales and courses was ~19 and ~19 yarns/10 mm for the wool fabric and ~17 and ~21 yarns/10 mm for the cotton fabric. The cotton fabric had an English cotton count of 1/40 and yarns were spun from combed cotton. The control fabric (a non-dyed 100% wool interlock) was used to ensure validity and reliability of instruments/tests. All fabrics were manufactured by Designer Textiles International Ltd., Auckland, New Zealand. Unless otherwise specified, for each property, five replicates of each fabric, 100 × 100 mm with different wale and course yarns were used.

The fabrics were washed to ensure removal of any remaining treatments used during yarn and fabric processing, and also to establish dimensional stability, following a published method [99] with modifications as per ISO 6330:2012 [100]. Detergent was used for one wash; thereafter, five washes were completed without detergent. A chemical pre-treatment was applied to the wool and cotton fabrics to increase hydrophilicity and uptake/fixing of the functionalizing agent. Wool was immersed in 0.05 mol/L potassium hydroxide in methanol for 15 min, and cotton immersed in 2 mol/L sodium hydroxide with deionized water for 30 min; both at ~20 °C. Not treated and pre-treated specimens were included in the present work.

All specimens were conditioned for 24 h at 20 ± 2 °C and 65 ± 4% RH in accordance with ISO 139:2005 [101]. Unless otherwise specified, all tests were carried out under these conditions.

### 2.2. Treatment for Conferring Electrical Conductivity and Encapsulation

Aqueous graphene ink DM-GRA-9003 (few-layer graphene 0.5–3 nm thick, 0.15–1 µm lateral diameter, 130 m^2^/g surface area) was purchased from Dycotec Materials Ltd., Swindon, UK. The non-cured ink has solids 1–2%, density 1 g/mL, viscosity >8cP at 2 °C, surface tension 24–30 mN/m, sheet resistivity 2–3 kΩ/ (40%T at 660 nm). The graphene ink was selected because it is a commercially available off the shelf product, readily available, and easy to apply to existing fabrics. Further, the product was designed to be cured at 120 °C which poses no risk to degradation of fabrics/yarns/fibers and is expected to yield better electrical conductivity and stability than reduced graphene oxide. Nano products were not used to avoid possible health implications (i.e., respiratory/dermal absorption).

Wool and cotton knit specimens 100 × 100 mm were immersed for 16 and 40 min, respectively, under ambient conditions and dried at 120 °C for 20 min in a Contherm Thermotec 2000 oven (Contherm Scientific Limited, Lower Hutt, New Zealand).

One of the three different encapsulation products was applied over the entirety of the 100 × 100 mm specimens. Additionally, graphene ink treated specimens of 50 × 50 mm were encapsulated in the center only (i.e., approximately 40 × 40 mm). Collectively referred to as functionalization, codes for each treatment type/encapsulation (encap) are given in Table 1.

SYLGARD™ 184 Silicone Elastomer Kit 4019862 0.5 KG (purchased from Dow^®^, Midland, MI, USA): Base and curing agent were mixed in a 10:1 ratio by volume as per the manufacturer’s instructions. The mixed solution was thinned with the solvent toluene in a 2:1 ratio (solvent:mixed polymer solution). A poly(dimethylsiloxane) polymer ([(CH_3_)3SiO]2Si(CH_3_)_2_), viscosity 1.0cSt at 25 °C (purchased from Sigma Aldrich^®^, 469319—50 mL; CAS 107-51-7) was also selected. The fabrics were immersed for 60 s, removed, placed on a wire gauze mat for 60 s to allow excess to drip off, and dried at 125 °C for 20 min in the oven.

Granger’s^®^ Clothing Repel was applied according to the product directions (“clean garment before use and lay flat, shake bottle, whilst garment is still wet/damp spray from a distance of 150 mm and leave for two minutes, remove any excess with a damp cloth and allow to dry naturally or tumble dry if the garment care label permits”). Fabrics were wetted by dropping five droplets of distilled water (0.08 mL) on the fabric surface (not cleaned) and left to dry flat overnight.

### 2.3. Fabric Structural Properties

Mass per unit area (g/m^2^) of the fabrics was determined following BS EN 12127:1998 [102] with a Mettler Toledo AT400 balance accurate to 0.001 g (Mettler-Toledo GmBh, Medic, Lower Hutt, New Zealand). Thickness (mm) of the fabrics was determined according to ISO 5084:1996 [103] with a digital thickness gauge (SDL Atlas MO34A) readable to 0.01 mm (Stockport, England). 

### 2.4. Moisture Related Properties

#### 2.4.1. Water Absorption

The time taken for a defined volume of distilled water (0.08 ± 0.01 mL droplet ISO 17617:2014 [104]) to absorb was determined. Water droplets were dropped on the fabric technical face from a height of 10 mm. The time (s) taken for the water droplet to completely absorb/adsorb in the fabric was measured (note that distinguishing absorption from adsorption was not possible). Water was considered absorbed/adsorbed when no visible water was apparent on the fabric surface. This method is similar to that previously adopted [105,106].

#### 2.4.2. Contact Angle

A sessile drop method was used to determine contact angle with a goniometer (±2° error). A 3-μL drop of deionized water was dropped on the technical face of the fabric (10 replicates) with a manually operated micrometer syringe, pressed down with a mechanical stage operated through the Fta32 Video software. Droplets were backlit with red illumination mitigating temperature changes. The camera (with zoom microscope) was positioned in front of the instrument (100 mm). Images were captured with the Fta32 Video software immediately after the droplet contacted the fabric surface. The instrument was calibrated with a 90° droplet slide. Conditions in which the instrument was located could not be controlled but were consistent throughout testing (measured with a tiny tag (Energy Engineering, Auckland, New Zealand)). 

#### 2.4.3. Moisture Regain

Moisture regain of the fabrics was determined by obtaining the oven dry mass following BS EN 12127:1998 [102]. Specimens were dried at 105 ± 3 °C (Contherm Thermotec 2000, Lower Hutt, New Zealand) for at least 40 min or until change in mass was less than 0.1% compared to the initial mass and weighed to obtain dry mass. Subsequently, for 24 h each fabric was stored at 20 ± 2 °C and 65 ± 4% RH and re-weighed. Moisture regain (%) was calculated by Equation (1).

m − mdry/mdry × 100(1)

where m is the mass of the conditioned specimen and mdry is the mass of the dried specimen.

#### 2.4.4. Permeability to Water Vapor

The water vapor permeability index of each fabric was determined following Appendix B of BS 7209:1990 [107] using a water vapor permeability tester (Campus Electronics and Mechanical, Otago School of Medicine, Dunedin, New Zealand) with a 90 mm diameter test area.

#### 2.4.5. Permeability to Air

Air permeability of the fabrics was determined following ISO BS EN 9237:1995 [108] with an air permeability tester (calibration certified ±5%, SDL Atlas Textile Testing Solutions, Stockport, UK) with a test area of 50 mm^2^. 

### 2.5. Electrical Conductivity

Electrical resistance (Ω) of the fabrics was measured with a two-probe digital multimeter (Digitech QM1544, Electus Distribution Pty, Ltd., Rydalmere, Australia). Measurements were taken parallel to the wales and courses of each specimen; diagonal measurements between the two corners were also taken for specimens with only a center strip of encapsulation. The fabric specimens remained without tensioning, with the digital multimeter, connectors, and fabric on a hard, flat surface. Electrical conductivity is reported, notwithstanding the common practice of reporting electrical resistance or resistivity. Electrical resistivity in Ω/m was determined by Equation (2).

ρ = Rwh/l(2)

where ρ is electrical resistivity in Ω/m, R is measured electrical resistance in Ω, w is width (meter), h is height (meter), and l is length (meter) of the specimen [57]. Electrical conductivity was reported in S/m by Equation (3).

σ = 1/ρ(3)

where σ is electrical conductivity in S/m and ρ is electrical resistivity in Ω/m [57].

### 2.6. Effects of Moisture on Electrical Conductivity

#### 2.6.1. Wetting

Three specimens (50 × 5 mm) were immersed in 10 mL depth of deionized water in a 90 mm diameter petri dish for 60 s. The fabrics were removed from the petri dish and placed on filter paper for 30 s to remove excess water. Mass (g) and electrical resistance (Ω) were measured before wetting, immediately after wetting, and every 5 min as the fabrics dried. The total time taken for fabrics to dry (equal mass to the initial state) was recorded. Following drying, fabrics were wet a second time and the experiment was repeated to provide evidence of sequential repeatability.

#### 2.6.2. Ambient Temperature and Humidity

Effects of changing temperature and humidity pertinent to next-to-skin end uses were examined. Two environmental conditions were selected: 35 ± 2 °C (controlled in Contherm Thermotec 2000 oven) with ~23% RH measured with a Tinytag Ultra data logger (Gemini Data Loggers; 0–95%, −30 to 50 °C; Energy Engineering, Auckland, New Zealand); 20 ± 2 °C and 65 ± 4% RH was controlled in a standard conditioned room.

Specimens (three 100 × 20 mm) were fastened between connectors of the two-probe digital multimeter taped to a poly(methyl methacrylate) board. The fabric specimens were elevated above the board, only contacting the connectors and surrounding air.

Electrical resistance parallel to the wales was first measured at 20 ± 2 °C and 65 ± 4% RH. Specimens were then transferred to 35 ± 2 °C and ~23% RH; electrical resistance was measured immediately and every minute thereafter for 15 min. Specimens were then returned to 20 ± 2 °C and 65 ± 4% RH, measured immediately and every minute thereafter for 15 min. Three repeat exposures in each environmental condition were taken. Response (%) was determined with Equation (4).

(4) $$\mathrm{Response}\;\left(\%\right)=\frac{\mathrm{Rc}-R0}{R0}\times 100$$

where Rc is electrical conductivity with exposure to temperature/humidity and R0 is electrical conductivity before exposure to temperature/humidity [109]. 

Recovery (%) was determined as per Equation (5).

(5) $$\mathrm{Recovery}\;\left(\%\right)=\frac{\mathrm{Rc}-\mathrm{Ra}}{\mathrm{Rc}-R0}\times 100$$

where Ra is electrical conductivity after recovery in standard conditions [110].

### 2.7. Durability in Use

#### 2.7.1. Effects of Wash

Durability to wash was tested with 1000-mL beakers on a DlAB MS-H-S10 analog magnetic hotplate stirrer 10-channel (DLAB Scientific Pvt Ltd., Los Angeles, CA, USA). For each fabric, three specimens were immersed individually in 400 mL of distilled water with a liquid detergent (>60% water, 10–20% anionic surfactant, <5% sodium chloride, <1% sodium hydroxide, <1% trisodium N-(2-hydroxyethyl) ethylenediaminetriacetate, <0.1% 1,2-benzoisothaizolin-3-one; pH 6.5–7.5), 40:1 water:detergent. Wash temperature was 30 °C and mechanical agitation was controlled at 300 rpm. The specimens were submerged within a few seconds and washed for 8 min. Parameters were selected based on specifications of gentle and wool wash cycles, and other simulated methods [29,111]. Specimens were rinsed in 30 °C distilled water for 30 s to remove residual detergent, dried flat on a drying rack overnight in ambient conditions. One hundred washes were carried out, drying at intervals of 1, 6, 10, 20, 30, 40, 50, 75, 100 washes. Following each interval, electrical conductivity was determined parallel to both the wale and course directions.

#### 2.7.2. Resistance to Abrasion

Resistance to abrasion was carried out in accordance with a modified version of ISO 12947-2:2016 [112] and ISO 12945-2:2000 [113]. Three specimens (90 mm diameter) of each treatment were rubbed with a 9 kPa abrasion weight to produce a load of 595 ± 7 g (specified for apparel and household textiles). The wool and cotton fabrics with no treatments were used as abradants (140 mm diameter) for functionalized wool and cotton, respectively. The selected number of cycles were 125, 500, 1000, 2000, 5000, 7000, 10,000, and every 5000 thereafter until 50,000 cycles were reached. Following each selected number of cycles, electrical conductivity was determined across the treated area parallel to the wale direction.

Optical microscope (Leica) images were taken of the fabrics and pills after 50,000 cycles. To count pills on the cotton specimen and abradant, a photographic image of the abraded area of the fabric was taken. A 15 × 15 mm section on the fabric was randomly selected and the number of pills counted using a cell counter plugin in Fiji [114]. The total number of pills which shed from the wool fabrics was counted. 

Images of pills were obtained with a Leica microscope at 6.3× magnification for pills on the cotton fabric and 2.5× magnification for pills from the wool fabric. Measurements were taken across the two axes of the pill with image software Fiji [114]. From the cotton specimens, 10 pills of each fabric were measured; while for wool each pill shed was measured. 

#### 2.7.3. Performance with Storage

Change in electrical conductivity over time was determined after storing specimens at 20 ± 2 °C, 65 ± 4% RH for 154 days. Electrical conductivity was determined parallel to the wale direction the same time each day for four weeks and then fortnightly thereafter for 154 days.

### 2.8. Statistical Analysis

Each fabric property was described by mean, standard deviation, and coefficient of variation. Statistical analysis was carried out using SPSS; a significance level of *p* ≤ 0.05 was accepted. Assumptions of normality and homogeneity were indicated by Levene’s test of equality with non-significant error variances (data not transformed) [115]. One-way ANOVA was used to determine significance of effects of the treatments on the various fabric properties. Tukey’s HSD multiple comparison post-hoc test was used to determine significance of differences for each comparison [115]. Repeated measure ANOVA was carried out for those tests for which repeated observations on specimens were required (i.e., wetting, exposure to different humidity/temperature, wash, abrasion, storage). Sphericity (equal variance among treatments) could not be assumed because Mauchly’s test of sphericity was violated, therefore the Greenhouse–Geisser test statistic was used.

## 3. Results

### 3.1. Fabric Structural Properties

Functionalization had an effect on mass and thickness of the wool (F_5,24_ = 566.07, *p* ≤ 0.01; F_5,24_ = 29.60, *p* ≤ 0.01, respectively) and cotton (F_5,24_ = 490.72, *p* ≤ 0.01; F_5,24_ = 21.74, *p* ≤ 0.01, respectively). A small increase in mass and thickness was evident with encap 0 (20 and −1% for wool, 26 and −7% for cotton, respectively) and when measured sequentially (wool: −0.3%; cotton: −0.7%). Mass and thickness increased with encap 1S (79 and 17% for wool; 48 and 7% for cotton, respectively). Encap 3G and encap 2P caused a decrease or minimal change in mass (wool: −6, −8%; cotton: −9, −15%, respectively) and thickness (wool: −8, −5%; cotton: −0.2, 2%, respectively).

### 3.2. Moisture Related Properties

For both wool and cotton, functionalization affected the time for water to be absorbed (F_5,4_ = 28.53, *p* ≤ 0.01; F_5,24_ = 165.25, *p* ≤ 0.01, respectively), contact angle (F_5,54_ = 46.78, *p* ≤ 0.01; F_5,54_ = 36.99, *p* ≤ 0.01), regain (F_5,24_ = 7.75, *p* ≤ 0.01; F_5,24_ = 8.44, *p* ≤ 0.01, respectively), permeability to water vapor (F_5,12_ = 4.52, *p* ≤ 0.05; F_5,12_ = 12.79, *p* ≤ 0.01, respectively), and permeability to air (F_5,54_ = 201.80, *p* ≤ 0.01; F_5,54_ = 30.86, *p* ≤ 0.01, respectively) (Table 2a,b). Encap 1S on wool and cotton was most different, having the greatest time for water absorption, contact angle, and lowest regain, permeability to water vapor, and to air (except for air permeability of cotton whereby encap 1S was highest). 

Images showing water droplet absorption over time are given in Figure S1. Spherical droplets formed, flattened, and were gradually absorbed bar not-treated cotton, which evaporated rather than absorbing. The cotton fabric which had not been treated was most similar to encap 1S for each property. Encap 3G often followed. Encap 0, encap 2P, and pre-treated specimens were similar whereby flattened droplets formed and absorbed. Encap 0 and encap 2P absorbed within ~2 s.

### 3.3. Electrical Conductivity

Fabrics which had not been treated were not electrically conductive (i.e., resistance could not be measured) and treating with graphene ink conferred electrical conductivity. Electrical resistance was read in ohms reflecting low resistance and high electrical conductivity: Up to 5 S/m (328 Ω) for wool and as high as 10 S/m (139 Ω) for cotton (Table 3 (a,b)).

Functionalization affected electrical conductivity of both wool and cotton (F_3,32_ = 45.95, *p* ≤ 0.01; F_3,32_ = 89.34, *p* ≤ 0.01, respectively); direction of measurement did not have a significant effect. The greatest decrease occurred with encap 1S, ~88% for both wool and cotton. Encap 3G had less of an effect on wool and cotton than encap 2P, 63 < 75%; 67 < 73% respectively; but variability with encap 3G was higher. Encap 0 measured sequentially reduced ~32 and 25% for wool and cotton, respectively.

Electrical conductivity of wool and cotton was also affected by whether or not this was a central strip of encapsulation (F_3,24_ = 4.31, *p* ≤ 0.01; F_3,24_ = 66.87, *p* ≤ 0.01, respectively) and the direction of measurement had a slight effect on wool (F_2,24_ = 3.79, *p* ≤ 0.05) but not on cotton. Encap 1S resulted in the greatest decrease in electrical conductivity followed by encap 2P and encap 3G for wool and cotton. Two groups were identified for measurement direction on wool (diagonal, courses; courses, wales).

### 3.4. Performance with Mositure

#### 3.4.1. Effects of Water

**Figure 1:**
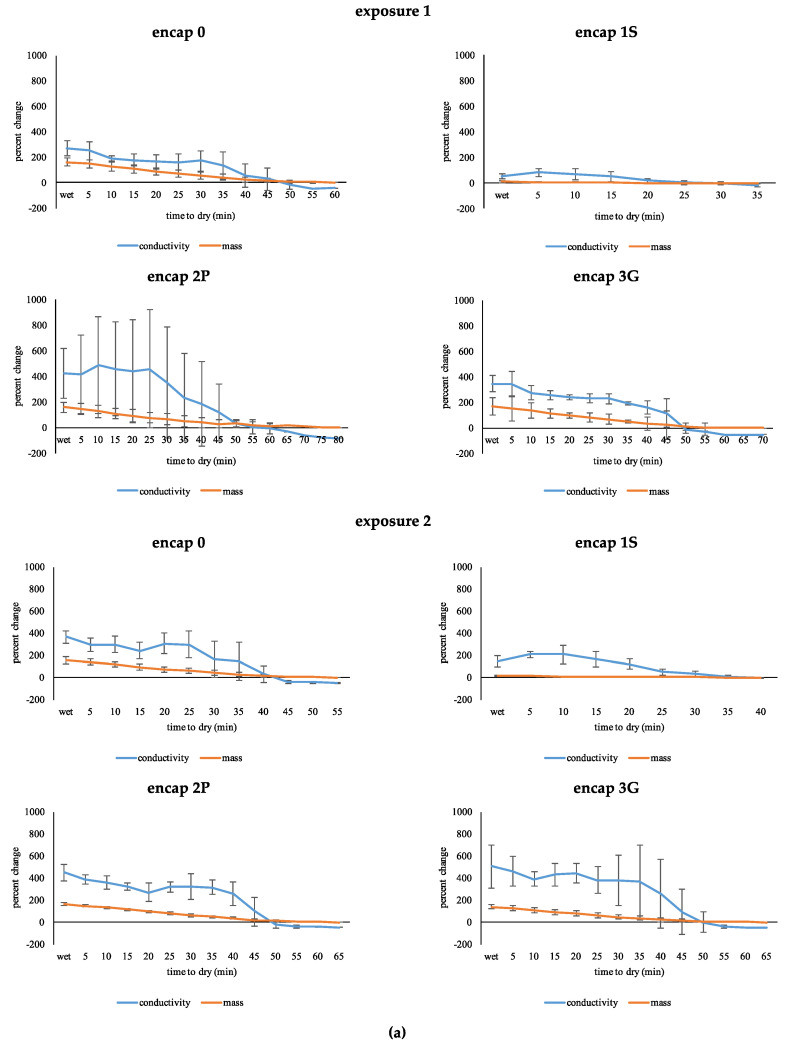

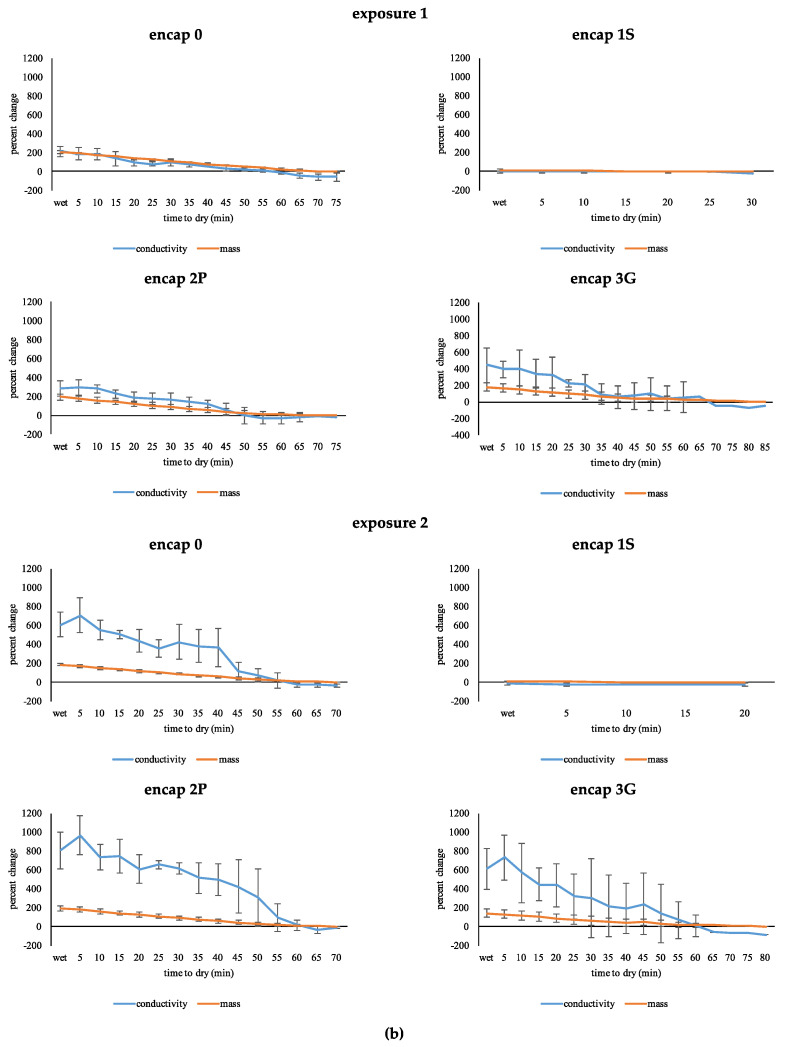
Effect of wetting on mass and electrical conductivity. (**a**) Wool. (**b**) Cotton.

Effects of wetting on functionalized fabrics are illustrated in Figure 1a,b. Some data points do not have standard deviations because specimens of the same treatment dried before others. Not surprisingly, wetting fabrics increased their mass (i.e., water was absorbed) and electrical conductivity increased; as fabrics dried, mass and electrical conductivity decreased again. The greatest change in mass and the most water absorbed typically resulted in the greatest increase in electrical conductivity, and the longest drying time.

For both fabrics, drying time of each exposure and each functionalization were not significantly different. After approximately 60 (wool) and 70 min (cotton), fabrics were dry. Mass and electrical conductivity differed with exposure for wool (F_1.15,9.21_ = 352.43, *p* ≤ 0.01; F_2.57,20.53_ = 66.50, *p* ≤ 0.01, respectively) and cotton (F_1.09,8.68_ = 170.18, *p* ≤ 0.01; F_2.20,17.56_ = 80.68, *p* ≤ 0.01, respectively). Functionalization altered each response for wool (F_3.46,9.21_ = 25.01, *p* ≤ 0.01; F_7.70,20.53_ = 5.76, *p* ≤ 0.01, respectively) and cotton (F_3.26,8.68_ = 16.39, *p* ≤ 0.01; F_6.59,17.56_ = 13.19, *p* ≤ 0.01, respectively). Encap 1S specimens showed minimal changes; the presence of some surface water could account for the increase.

For wool, at 45 min was when electrical conductivity was most similar to that of the initial state; the same occurred for cotton at approximately 50 to 60 min. Rate of change in electrical conductivity was similar for sequential measurements close to the beginning (5 to 25 min) and end (50 to 80 min); however, fewer values were recorded for the latter as some fabrics had already dried. After drying, electrical conductivity reduced below the initial state.

The response in mass between each exposure did not differ for wool but did differ very slightly for cotton (F_1.00,8.00_ = 8.23, *p* ≤ 0.05), and functionalization did not affect this response. A difference was identified for electrical conductivity of wool and cotton between exposures (F_1.00,8.00_ = 20.91, *p* ≤ 0.01; F_1.00,8.00_ = 14.51, *p* ≤ 0.01, respectively) of which functionalization did not effect. Therefore, the response was not reproducible for sequential exposures.

#### 3.4.2. Effects of Environmental Temperature and Humidity

**Figure 2:**
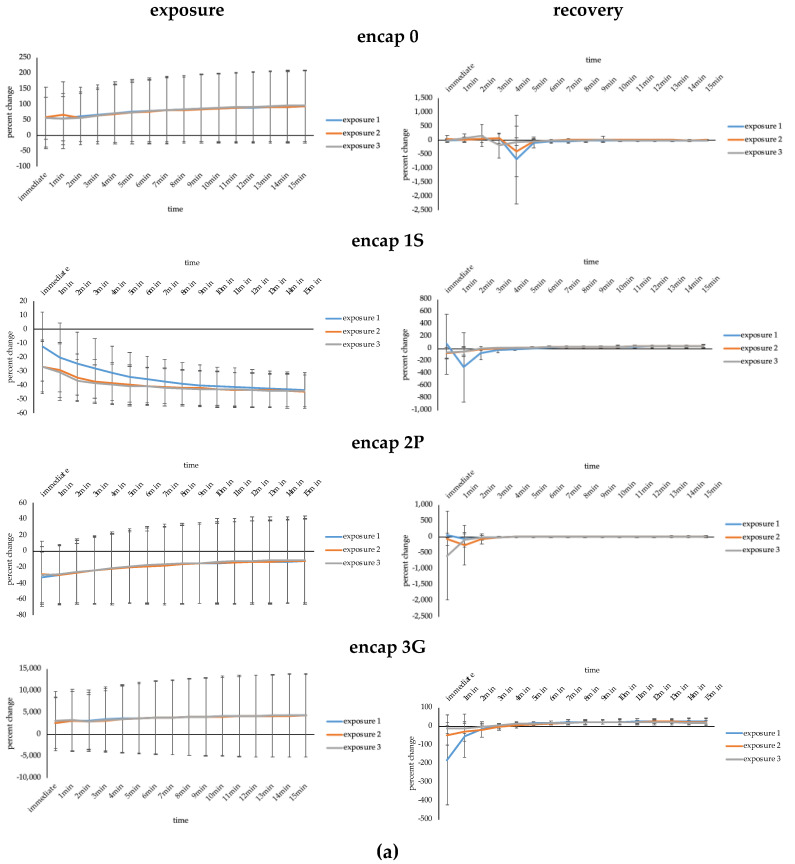

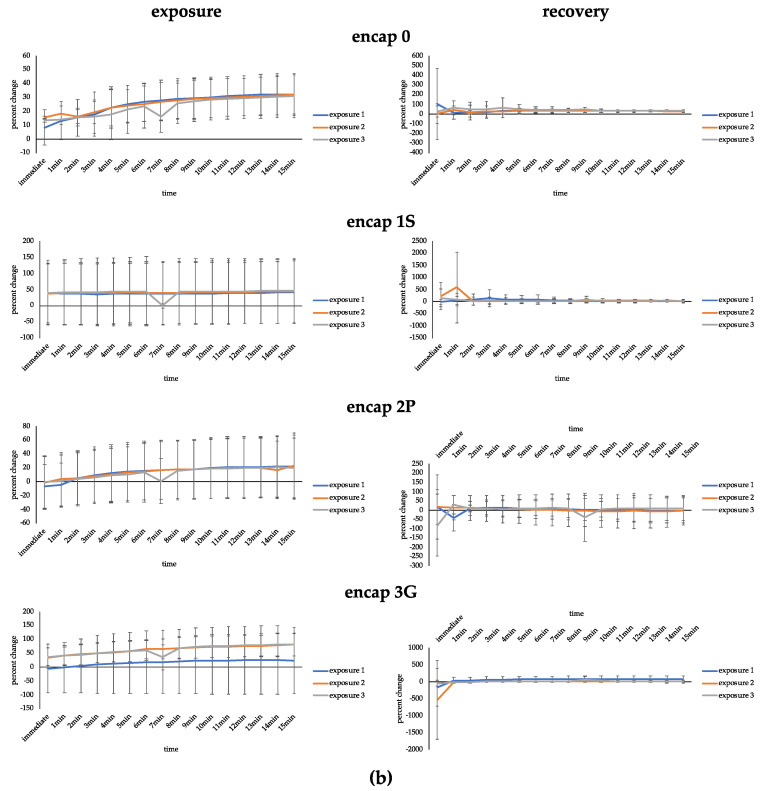
Effect of temperature and humidity on electrical conductivity. (**a**) Wool. (**b**) Cotton.

Performance with exposure and recovery to different ambient temperatures and relative humidity is given in Figure 2a,b. The pattern of change was similar for each of the three sequential exposures and functionalization had no significant effect on this response for wool or cotton. The response in electrical conductivity differed over 15 min of exposure for wool and cotton (F_1.04,8.28_ = 7.79, *p* ≤ 0.05; F_1.90,15.23_ = 61.06, *p* ≤ 0.01, respectively) and functionalization had an effect on the pattern of response (F_3.10,8.28_ = 6.23, *p* ≤ 0.05; F_5.71,15.23_ = 7.47, *p* ≤ 0.01, respectively). Encap 1S was most different, especially compared to encap 0 and encap 3G when comparing functionalization treatments for wool and cotton specimens. For wool, change was increasingly negative for encap 1S, showing opposite effects to remaining functionalized specimens (encap 2P also had a negative change but showed low change with increasing time). Whereas encap 1S use for cotton resulted in minimal responses to temperature and humidity fluctuations.

Recovery for each of the three sequential exposures showed similar trends (i.e., there was no significant difference for wool or cotton), and functionalization did not significantly change the response for either fabric. For wool and cotton, the response significantly changed over 15 min (F_1.06,8.44_ = 9.37, *p* ≤ 0.01; F_1.76,14.09_ = 12.94, *p* ≤ 0.01, respectively) but there was no effect of functionalization. In general, electrical conductivity did not return to the starting level after the 15 min recovery period.

Recovery was also determined after 24 h. For wool and cotton, there was no significant difference between the starting electrical conductivity of the fabrics and that measured after 24 h and each functionalization was relatively similar. 

### 3.5. Durability in Use

#### 3.5.1. Wash

**Figure 3:**
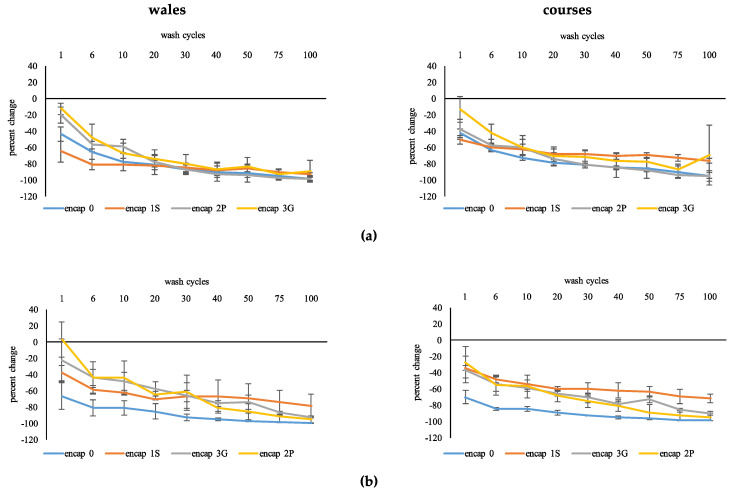
Performance of functionalized fabric with wash. (**a**) Wool. (**b**) Cotton.

Wash had a significant effect on electrical conductivity of wool (F_1.12,17.96_ = 40.35, *p* ≤ 0.01) and cotton (F_1.11,17.73_ = 81.95, *p* ≤ 0.01) causing a reduction as number of washes increased (Figure 3a,b). Effects were likely attributable to graphene removal from fabrics, from the change in color of the wash solution and decreased intensity in color of specimens. The pattern of change in electrical conductivity differed among functionalization treatments for wool and cotton (F_3.37,17.96_ = 11.13, *p* ≤ 0.01; F_3.32,17.73_ = 21.19, *p* ≤ 0.01, respectively) but no effect was found based on direction of measurement. Therefore, encapsulants reduced what appeared to be loss of graphene ink from the fabric to the wash liquor, especially for encap 1S. For both wool and cotton, encap 0 was different to encapsulants but effects of the three encapsulants were comparatively similar. Notwithstanding in some instances, encap 1S had the smallest change, followed by encap 3G and encap 2P. The extent of change decreased as the number of wash cycles increased: Wash cycles 20 and 30; 40 and 50 were most similar.

#### 3.5.2. Abrasion

##### Electrical Conductivity

**Figure 4:**
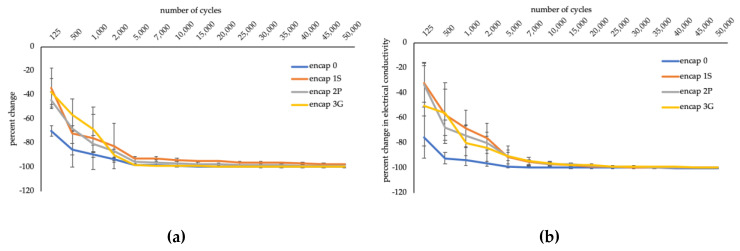
Performance of functionalized fabrics with abrasion. (**a**) Wool. (**b**) Cotton.

Electrical conductivity of wool and cotton decreased with exposure to abrasion (F_1.50,12.02_ = 47.71, *p* ≤ 0.01; F_2.08,16.61_ = 112.03, *p* ≤ 0.01, respectively), and continued to decrease with increased cycles (Figure 4). The reduction may be a result of graphene removed/transferred (i.e., crocking) over the specimens and to the abradant. Both encapsulant and graphene ink could be removed. Alternatively, the encapsulant may remain but become disrupted allowing graphene ink to be dislodged and transferred around the specimen and/or the abradant. Transfer of functionalization from specimens to abradants was not sufficient to confer electrical conductivity to the abradants while all specimens retained an acceptable level of electrical conductivity.

Additionally, Figure 4 suggests encapsulated specimens had a more gradual rate of decrease than encap 0; the rate of change declines after approximately 5000 cycles for wool and 7000/10,000 for cotton. For wool, encap 1S typically retained the highest electrical conductivity followed by encap 2P, encap 3G, and encap 0. Encap 3G was typically highest for cotton, followed by encap 2P, encap 1S, and encap 0. 

##### Pilling

**Figure 5:**
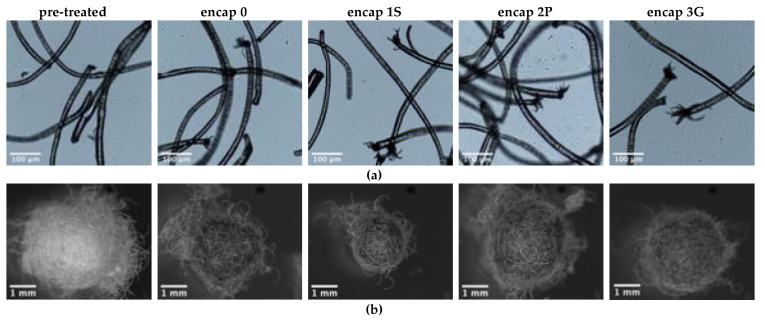
Effect of abrasion (50,000 cycles) on wool fibers. (**a**) Shed wool fibers. (**b**) Shed pills.

Microscope images of fibers shed from the wool fabrics are given in Figure 5a,b, and images of fabrics are given in Figure S2. Wool fabrics only pilled early in the exposure and break off, thereafter fibers continued to be shed. Encap 1S performed differently in that the encapsulant was visible in crocking and shed fibers, and less presence of surface fibers remained on specimens. Shed fibers had frayed ends, suggesting they were broken rather than pulled from fabrics. Fibers from functionalized specimens were more frayed and split than those from pre-treated fabrics.

The number of pills shed from wool fabrics differed based on functionalization. The greatest number (total from three specimens) of pills was shed from encap 0 fabrics (13), while the remaining fabrics ranged between 9 and 6: Pre-treated (7), encap 1S (9), encap 3G (6), encap 2P (7). Thus, graphene ink may contribute to increased pill formation and shedding, whereas encapsulation may result in a decrease. Each axis of the pills differed (F_1,74_ = 8.05, *p* ≤ 0.01) and functionalization had an effect on the dimensions of pills (F_4,74_ = 4.68, *p* ≤ 0.01) (Table S1). Pills of pre-treated specimens had the greater dimensions than those of functionalized fabrics.

**Figure 6:**
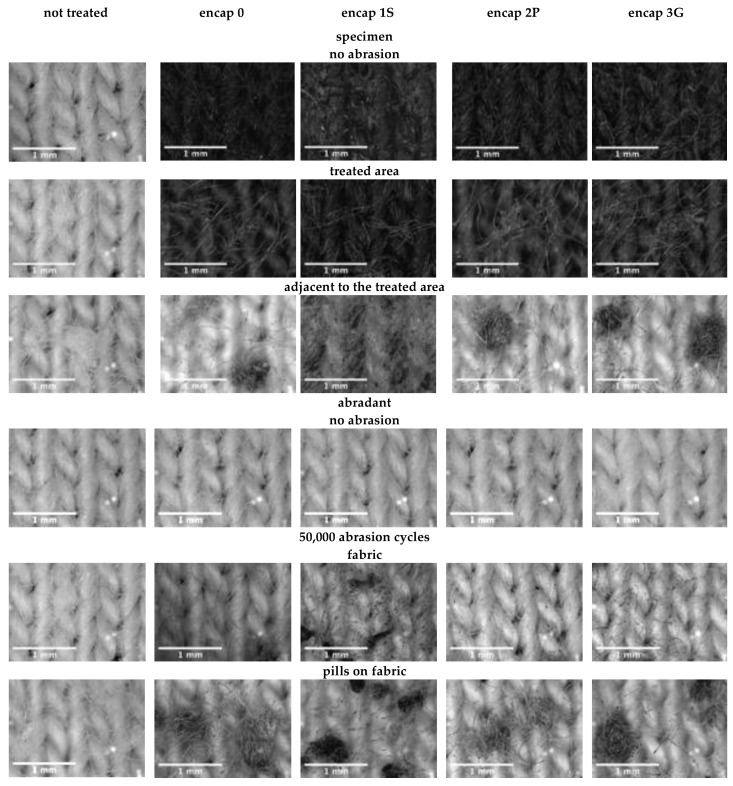
Effect of abrasion on cotton fabric surface.

Pills on cotton were dense, present on the functionalized area, adjacent to this, and on abradants. The number of pills differed between specimens and abradants (F_1,20_ = 333.34, *p* ≤ 0.01; Figure 6, Table S1); therefore, each fabric was examined separately: Functionalization had an effect on pilling of fabric specimens (F_4,10_ = 16.64, *p* ≤ 0.01) and abradants (F_4,10_ = 16.28, *p* ≤ 0.01). Pre-treated specimens had the greatest density, similar to encap 2P. As expected, pre-treated specimens only had cream pills. Thus, the color of the pills on functionalized specimens can be attributed to graphene ink and encapsulants. Encap 1S had the most different effect, while encap 3G, encap 0, and encap 2P were similar.

Pills on the treated area were least visible and dense, whereby fuzzing (i.e., fibers extending from yarns) was evident and/or the pills were re-distributed or shed. Pill formation was minimal on adjacent areas of encap 1S specimens, each of the other specimens had pill formation in this area. Spreading of the encapsulants during treatment application may have affected pill formation (i.e., prevent or encourage fiber entanglement). Crocking and tangling of graphene ink and encapsulated fibers was evident on abradants. The greatest transfer occurred with encap 1S and the pills of encap 1S specimens differed from the other fabrics: The encapsulant was evident with pills being tighter, smaller, and darker. 

Dimensions of pills were different based on axis of measurement (F_1,90_ = 22.57, *p* ≤ 0.01) and functionalization (F_4,90_ = 18.28, *p* ≤ 0.01). Encap 1S and pre-treated fabrics had the smallest and largest pills, respectively. Pill dimensions of the abradant were also affected by the measurement axis (F_1,90_ = 12.18, *p* ≤ 0.01) and functionalization (F_4,90_ = 7.44, *p* ≤ 0.01). Pills from abradants of encap 1S were smaller than the other treatments, which were similar.

#### 3.5.3. Storage

**Figure 7:**
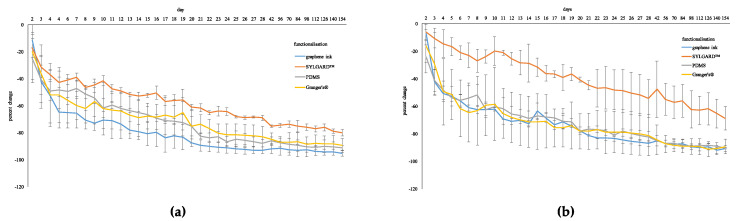
Performance of functionalized fabrics with storage. (**a**) Wool. (**b**) Cotton.

A significant effect of time on electrical conductivity of wool and cotton fabrics was identified (F_1.19,9.48_ = 17.05, *p* ≤ 0.01; F_3.25,25.99_ = 80.05, *p* ≤ 0.01, respectively), decreasing over time (Figure 7). Functionalization effected this response (F_3.56,9.48_ = 4.49, *p* ≤ 0.05; F_9.75,25.99_ = 11.58, *p* ≤ 0.01, respectively). For wool and cotton, all fabrics had a greater initial decrease (i.e., first 28 days) and then showed a decrease in the extent of change with sequential measures, with some indication of plateauing. However, electrical conductivity continued to decrease over time.

Encap 0 had a greater initial decrease compared to encapsulated specimens and generally attributed with the greatest change in electrical conductivity throughout the testing period. Encap 1S showed the smallest change in electrical conductivity, followed by encap 3G and encap 2P.

So, to consider these results together, efficacy in treatments and their effects are ranked in decreasing order of desired effect (Table 4). The lowest level of electrical conductivity (encap 1S) can still be considered acceptable. Encap 1S is also most stable with wetting, fluctuating temperature and humidity, and durability to wash, abrasion, and storage.

## 4. Discussion

### 4.1. Moisture Transfer

Scientific reports on changes with functionalization/encapsulation to transfer of moisture pertaining to thermoregulation are sparse. The desired performance depends on the application; however, all next-to-skin applications relate to maintaining homeostasis [2,3]. For most apparel end-uses, fabrics which allow passage of water vapor from a garment microclimate to the ambient environment or within the textile structure followed by evaporation are desirable. Wool and cotton knits are often viewed as having these properties, and single jersey structures have greater permeability to permit transfer, compared to other common knit structures (e.g., rib, interlock) [116]. Retaining such properties following functionalization is important for fabrics worn on the body.

Hydrophobicity [50,117,118] and hydrophilicity [119] of wool fabrics has been indicated by the water contact angle and pre-treatments can increase hydrophilicity [50]. Cotton is typically attributed with hydrophilicity, indicated by small contact angles [8,15,31,53]. The contact angles of functionalized surfaces can differ depending on the underlying surface [120] and composition of the droplet [121]. No evidence for contact angle of graphene functionalized wool was found; that of cotton stipulated an increased contact angle following functionalization with reduced graphene oxide [8,15,21,31,33,122]. In the present work, a graphene ink was used, perhaps explaining the difference in the contact angle compared to published research (i.e., not measurable with graphene ink due to rapid uptake of the water droplets). Effects on water absorption support this finding.

Additionally, information pertaining to effects of encapsulation on contact angle of graphene functionalized fabrics was difficult to identify. Two of the three encapsulations in the present work yielded surfaces with higher contact angle indicating an extent of hydrophobicity and therefore water repellence/proofing. Water absorbency time also increased, confirming this result. Therefore, repellence of water and contaminants may be evident with encapsulation.

With graphene ink, decreased regain and increased permeability to water vapor and to air of wool, compared to a decrease in all three properties for the cotton, is likely due to differences in deposition patterns. Cotton fabric yarns were closely interlocked/aligned for which graphene can readily deposit due to the high surface area, whereas with comparatively large interstitial spaces, graphene ink may have passed through interstitial spaces in wool rather than attaching to yarns/fibers. An example of similar changes in published research was found. Permeability to air (ASTM D737-04:2004), mean pore diameter (ASTM D6767-14:2014), and water vapor permeability (ISO 8096:2005) of woven and knit cotton fabrics decreased following immersion in graphene oxide solution and subsequent reduction reaction, resulting in 0.75, 1.5, and 2.25% on the weight of the fabric [16].

Decreased regain, permeability to water vapor, and to air (bar air permeability of cotton) observed with encap 1S could be linked with increased fabric thickness and mass due to a thick cohesive layer that formed on the fabric. Thicker fabrics generally resist transmission of heat, water vapor, and air [3]. The presence of air principally determines thermal and moisture transfer because the performance of the different fiber types is similar [2,3]. More material (i.e., fabric, graphene, encapsulant) and air spaces beneath the encapsulant or within interstitial spaces/fibers may be trapped, increasing matter and air present to move through. Penetration of the encapsulants within yarns/fibers changing composition and effective diameter could also contribute to differences [73]. Differences in wetting show the same patterns (i.e., increased hydrophobicity may relate to decreased transfer of moisture and air); this was particularly evident with encap 1S. Thus, encap 1S may have limited acceptability for large sections of next-to-skin apparel, although may be acceptable for smaller patches. Published research findings were similar to the present work. Decreased air permeability (ISO BS EN 9237:1995), water vapor permeability (cup method), and water retention (immersion) were reported following surface treatment of microporous polyurethane and fluorocarbon of two groups of electrically conductive three-layered interlock fabrics (67% cotton/33% polyester, carbon core polyester filament, and hollow polyester yarns with polypropylene yarns, or 80% polyester/20% stainless steel yarns) [46].

### 4.2. Electrical Conductivity

#### 4.2.1. Encapsulation

Electrical conductivity was higher (i.e., electrical resistance lower) than that of published research on encapsulating graphene-treated fabrics or encapsulation of other functionalized fabrics with poly(dimethylsiloxane) products (Table 5). Dissimilarities in fabric construction, graphene constituents, and encapsulation composition were recognized possibly contributing to observed differences. 

Penetration of encapsulants through graphene ink, in the fabric interstitial spaces, among yarns, fibers, and within fibers contributed to observed decreases in electrical conductivity [25,73,78]. More viscous encapsulants reduced penetration but thickness became greater (i.e., double thickness and increased density has been reported [73]). Reduced contact between electrically conductive surfaces and connectors resulted, also contributing to decreased electrical conductivity. Applying a central strip of the encapsulants permitted contact between the connectors and the graphene ink-treated surface, rather than the encapsulant layer. Electrical conductivity was still lower; thus, differences with each of the three encapsulants can be attributed to both effects. Variability between taking sequential measurements (i.e., that of encap 0) could also account for some of the observed differences. 

#### 4.2.2. Effects of Wetting and Changes in Environmental Temperature and Humidity

Electrical conductivity has been reported to increase with exposure to moisture. Researchers suggest water molecules dope graphene [123], produce protonation and density charge of the carriers [124], or enhance polarization and increase the dielectric constant [125,126,127]. Higher levels of humidity produce a continuous surface water layer which increases electrical conductivity [128]. Thus, exposure to water will have a more pronounced, rapid effect on electrical conductivity, as evident in the present work. Effects of various humidity levels on electrical conductivity have been investigated, however, effects of exposure to wetting are seldom included in published research. Wetting occurs from several sources while the fabric is worn (i.e., perspiration, rain, liquid spills, or for more specialized applications such as sensing with swimmers or firefighters entering a building with activated sprinklers).

The response as the fabric dries is also critical. Although fabrics return to their initial dry state, electrical conductivity will not necessarily match the starting level, nor respond consistently with repeated exposures. Minimal change with exposure to water is desirable if the functionalized fabric is intended to detect factors other than the moisture presence; with this requirement, encap 1S performs best. Predictable change may also be acceptable and manageable. The pattern for the extent of change can be likened to water absorbency time and contact angle of the encapsulated fabrics.

#### 4.2.3. Durability in Use

All fabrics retained electrical conductivity following 100 washes to an “acceptable” level; the trend of decrease was also predictable. Examples of studies demonstrating wash performance of encapsulated functionalized fabrics are given in Table 6. Performance in the present work compared well with other scientific reports. 

Greater release of graphene ink in wash liquor decreases connections to form conductive networks. Ideally, encapsulants increase fixation of graphene ink by producing a barrier between the graphene ink treated surface and any external exposure. However, graphene ink loss could also increase if the graphene ink and encapsulant are adhered together but not effectively fixed to the fabric. Decrease in electrical conductivity was less with all encapsulants, least with encap 1S; therefore, the cohesive coverage likely prevented graphene ink removal. 

Despite apparent minimal change to the encapsulant layer itself, degradative changes to the underlying functionalized components and therefore electrical conductivity can still occur. This was evident in two studies [73,80]. No degradation, peeling, or cracking of the encapsulants occurred, rather decreased electrical conductivity was suggested to relate to increased resistance between silver-coated polyamide and electrodes under the encapsulants [80]. 

No scientific literature pertaining to the effects of abrasion on encapsulated functionalized fabrics was found. Decreased electrical conductivity likely relates to crocking, removing graphene and therefore reducing potential conductive networks. The presence of pills may also disrupt transmission of electrical signals; therefore, after reaching a certain pill density, effects on electrical conductivity may plateau. 

Pills fixed to fabrics were most critical on the cotton fabrics. Lower pill density and dimensions on functionalized areas could relate to adhesion between yarns and fibers restricting fiber extraction and entanglement. Black pills formed adjacent to the functionalized area and on abradants suggesting fibers from functionalized specimens were caught in pills or treatments transferred due to crocking. 

Shed fibers were most evident on wool fabrics. Fibers shed from pre-treated fabrics had sharp transverse breaks, transverse cracks, and/or granular fracture, whereas those of functionalized fabrics were fibrillated, had breaks with multiple splitting, axial splits, surface wear, and/or break with a bushy end [129]. Multiple splitting led to breakage, which may have later become a more rounded shape [129] that could have occurred with pre-treated specimens; graphene ink and encapsulants may have contaminated fibers, preventing rounding. Rounded breaks may be more acceptable related to wear properties of fibers remaining on fabrics.

Decreased electrical conductivity with storage, particularly over the first 28 days, could relate to fastening and removing the connectors. Additionally, graphene not effectively bound may have been removed on the first few days, thereafter, being relatively stable. Changes could also be a result of degradation due environmental conditions (i.e., 20 °C and 65% RH) (i.e., moisture presence). Exposure to light was minimized by storing specimens in the dark, therefore, degradation from light did not occur.

Both of these fabrics could be used in apparel sensing applications in work safety, sporting, or health care. For example, in applications where temperature/humidity fluctuates or there is exposure to water, the use of SYLGARD™ 184 (especially on graphene treated cotton single jersey) would be best suited, as electrical conductivity would remain relatively stable, but may change with exposure to other agents (i.e., strain, gas/volatile exposure). However, while this treatment resulted in some changes to structural properties, moisture related properties, and permeability to air (less desirable for wear next to the skin), a small patch/area on a garment would likely be acceptable. Where retaining properties of the original fabric is a priority (i.e., large dimension, sensitive next-to-skin areas), some compromises in performance (sensor interference, durability) may be needed (e.g., use of graphene ink only, poly(dimethylsiloxane), and Granger’s^®^ Clothing Repel). These treatments have potential for water and/or temperature and humidity sensors. Additionally, if the change in electrical conductivity with exposure to a parameter (e.g., strain, gas/volatiles) is the opposite to the effect of temperature and humidity (could be the case for wool with SYLGARD™ 184), then the fabric could possibly function as a dual sensor.

## 5. Conclusions

Encapsulation with poly(dimethylsiloxane) of graphene ink functionalized wool and cotton single jersey changed performance of the fabrics. Effects of the encapsulants were consistent among properties, especially with use of SYLGARD™ 184 resulting in the greatest change (i.e., electrical conductivity, mass, thickness, water absorbency time, contact angle, regain, permeability to water vapor and to air) and a fabric most dissimilar to the original fabric.

Electrical conductivity was maintained with encapsulation, demonstrated with exposure to various external elements. The performance of SYLGARD™ 184 in terms of durability to in-use processes and with exposure to water was superior, but changes with different environmental ambient temperature and humidity were observed, which were undesirable when sensing other elements. Durability was conferred with each of the encapsulants, and to a greater extent than previously published research, although further improvements seem feasible (e.g., minimise graphene and encapsulant removal and therefore retain electrical conductivity). Effects of the functionalizing treatments on extension/recovery of the fabric, and also on bending, warrant investigation where the end application requires these properties.

## Figures and Tables

**Table 1:** Codes for functionalized fabrics.

Functionalization	Graphene Ink Only	SYLGARD™ 184	Poly(dimethylsiloxane)	Granger’s^®^ Clothing Repel
code	encap 0	encap 1S	encap 2P	encap 3G

**Table 2:** Effects of functionalization on fabric moisture transfer and permeability to air.

**(a) Wool**						
	**Not Treated**	**Pre-Treated**	**Encap 0**	**Encap 1S**	**Encap 2P**	**Encap 3G**
**water absorbency time (s) n = 5**						
mean	94.80	11.40	2.80	300.00	3.00	160.60
(s.d., CV%)	(32.52, 34.31)	(1.82, 15.94)	(0.45, 15.97)	(0.00, 0.00)	(0.00, 0.00)	(117.14, 72.94)
**contact angle (°) n = 10**						
mean	11.58	79.20	0.00	96.63	0.00	58.50
(s.d., CV%)	(17.24, 148.86)	(14.37, 18.14)	(0.00, 0.00)	(17.99, 18.61)	(0.00, 0.00)	(38.78, 66.29)
image	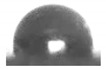	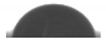	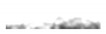	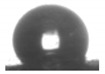	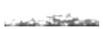	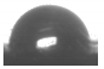
**regain (%) n = 5**						
mean	13.52	13.80	10.22	5.43	11.71	12.40
(s.d., CV%)	(1.04, 7.73)	(1.06, 7.65)	(5.18, 50.69)	(2.59, 47.77)	(1.08, 9.18)	(0.58, 4.68)
**water vapor permeability index n = 3**						
mean	104.56	92.31	96.06	78.24	101.84	102.50
(s.d., CV%)	(95.09, 91.74)	(7.58, 8.21)	(0.71, 0.74)	(12.21, 15.61)	(6.21, 6.10)	(5.08, 4.96)
**air permeability (mm/s) n = 10**						
mean	811.62	948.56	1028.72	304.60	1275.88	928.52
(s.d., CV%)	(47.37, 5.84)	(82.12, 8.66)	(84.50, 8.21)	(48.45, 15.90)	(71.81, 5.63)	(85.95, 9.26)
**(b) Cotton**						
	**Not Treated**	**Pre-Treated**	**Encap 0**	**Encap 1S**	**Encap 2P**	**Encap 3G**
**water absorbency time (s) n = 5**						
mean	300.00	300.00	2.60	300.00	2.40	205.80
(s.d., CV%)	(0.00, 0.00)	(0.00, 0.00)	(0.55, 21.07)	(0.00, 0.00)	(0.55, 22.82)	(62.24, 30.24)
**contact angle (°) n = 10**						
mean	67.93	90.55	0.00	75.24	0.00	43.92
(s.d., CV%)	(6.64, 9.78)	(6.11, 6.75)	(0.00, 0.00)	(11.74, 14.84)	(0.00, 0.00)	(47.38, 107.88)
image	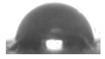	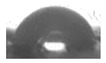	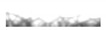	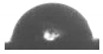	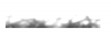	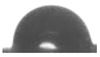
**regain (%) n = 5**						
mean	7.94	9.08	8.62	4.53	7.27	8.48
(s.d., CV%)	(2.78, 35.08)	(0.50, 5.54)	(0.76, 8.80)	(0.60, 13.15)	(0.67, 9.20)	(0.56, 6.66)
**water vapor permeability index n = 3**						
mean	106.53	231.66	219.82	202.47	209.99	258.74
(s.d., CV%)	(2.68, 2.52)	(22.21, 9.59)	(14.14, 6.43)	(26.68, 13.18)	(15.89, 7.57)	(46.35, 17.91)
**air permeability (mm/s) n = 10**						
mean	377.75	363.72	285.56	444.88	404.80	305.60
(s.d., CV%)	(20.07, 5.31)	(10.61, 2.92)	(26.40, 9.25)	(58.08, 13.06)	(36.34, 8.98)	(32.81, 10.74)

**Table 3:** Effect of functionalization on electrical conductivity (S/m).

	**Wales**				**Courses**			
	**Encap 0**	**Encap 1S**	**Encap 2P**	**Encap 3G**	**Encap 0**	**Encap 1S**	**Encap 2P**	**Encap 3G**
	**(a) Wool**							
	**“full” encapsulation**							
mean	3.18	0.28	0.53	0.23	2.88	0.34	0.69	0.30
(s.d., CV%)	(1.17, 36.81)	(0.13, 44.57)	(0.18, 34.05)	(0.17, 73.90)	(1.06, 36.69)	(0.14, 41.32)	(0.64, 93.08)	(0.22, 64.18)
	**center strip of encapsulation**							
mean	2.62	1.06	1.16	1.18	2.01	0.92	1.18	1.31
(s.d., CV%)	(1.16, 44.24)	(0.52, 49.19)	(0.40, 34.04)	(0.67, 56.83)	(0.39, 19.28)	(0.56, 60.68)	(0.74, 62.73)	(0.61, 46.88)
	**(b) Cotton**							
	**“full” encapsulation**							
mean	7.65	0.92	1.63	2.38	6.58	1.12	1.52	2.37
(s.d., CV%)	(2.14, 28.00)	(0.34, 36.54)	(0.32, 19.97)	(0.15, 6.16)	(1.33, 20.17)	(0.37, 32.62)	(0.24, 16.06)	(0.41, 17.27)
	**center strip of encapsulation**							
mean	19.02	3.40	6.69	7.69	19.03	3.31	7.17	6.36
(s.d., CV%)	(4.32, 22.73)	(1.73, 50.71)	(0.49, 7.29)	(2.26, 29.41)	(2.71, 14.22)	(1.47, 44.25)	(0.76, 10.57)	(1.05, 16.52)

**Table 4:** Efficacy of treatments ranked for effects on electrical conductivity.

	Encap 0	Encap 1S	Encap 2P	Encap 3G
**(a) Wool**				
electrical conductivity	1	4	2	3
change with wetting *	2	1	3	4
change with temperature/humidity *	2	1	3	4
wash	4	1	2	3
abrasion	4	1	2	3
storage	4	1	2	3
**(b) Cotton**				
electrical conductivity	1	4	3	2
change with wetting *	2	1	3	4
change with temperature/humidity *	2	1	3	4
wash	4	1	3	2
abrasion	4	1	2	3
storage	4	1	2	3

1 most desirable, 4 least desirable; * opposite order if sensor for water or temperature/humidity is sought.

**Table 5:** Electrical conductivity of graphene treated and poly(dimethylsiloxane) functionalized fabrics.

Fabric, Reference	Functionalization	Encapsulation	Electrical Conductivity/Resistance
100% wool and 100% cotton single jersey the present study	graphene ink	SYLGARD™ 184, poly(dimethylsiloxane), Granger’s^®^ Clothing Repel	wool: 5.02 S/m (327.66 Ω) cotton: 10 S/m (138.64 Ω); decrease to 0.28 S/m (5.54 Ω), 0.92 S/m (1.60 Ω) (SYLGARD™ 184); 0.53 S/m (2.53 Ω), 1.63 S/m (0.86 Ω) (poly(dimethylsiloxane)); 0.23/S/m (13.38 Ω), 2.38 S/m (0.58 Ω) (Granger’s^®^)
100% cotton weave 90° or 45° angle between vertical and horizontal yarns; yarn count, Ne 40 s; density 60 × 60 two samples – 60 × 60 mm × 0.2 mm^3^; 30 × 10 mm × 0.46 mm^3^ [30]	dipped in multilayer graphene nanosheets 4 mg/mL dispersion, three cycles	SYLGARD™ 184, immersion, cured 100 °C 30 min	0.21 Ω, 0.49 Ω to 0.26 Ω, 0.68 Ω for 90° and 45° fabrics, respectively fabrics due to encapsulation
100% cotton and 100% wool weft knit, 0.55, 0.7 mm thickness, 223.9, 509.7 µm fiber diameter, 0.22, 0.38 kg/m^2^ area density, respectively, “dog bone” shape 100 × 6 mm [25]	graphene nanoparticles and carbon black dispersed in deionized water and sodium dodecylbenzene-sulfonate	Ecoflex^®^ (0030), liquid elastic elastomer, cured 90 °C 45 min, fabric placed in this layer and non-cured Ecoflex^®^ on top, cured; yield 4 mm thickness	surface resistance ~286.54 Ω and 232.15 Ω for cotton and wool; increased to 1.95 KΩ and 1.22 KΩ with the Ecoflex^®^ layer, respectively
polyurethane sponge [54]	immersion in carbon nanotubes	half cured (70 °C, 20 min) poly(dimethylsiloxane)	electrical resistance after first dip 450 KΩ and five treatments 2.3 KΩ
polyurethane sponge [55]	nickel nanoparticles and graphene	poly(dimethylsiloxane) upper and lower layer, half cured	electrical resistance 2.5 MΩ with one cycle, 29.72 KΩ after five cycles

**Table 6:** Performance of encapsulated functionalized fabrics with wash.

Fabric, Reference	Functionalization	Encapsulation	Performance with Wash
100% wool and cotton single jersey the present study	graphene ink	SYLGARD™ 184, poly(dimethylsiloxane), Granger’s^®^ Clothing Repel	graphene ink, encap 1S, encap 2P, encap 3G following 100 washes show decline: Wool 98, 93, 89, 98% (wales); 95, 77, 69, 94% (courses); cotton 99, 78, 92, 95% (wales); 99, 72, 90, 94% (courses), respectively
100% cotton fabric [79]	surface mount LEDs soldered to stitch traces, silver conductive yarn	Gear Aid Sil-Net™ silicon seam sealer	0.38 Ω/m following 16.67 h washing and drying (Whirlpool^®^ Ultimate Care II washing and tumble-drying machines)
woven fabric [72]	miniaturized embroidery circuits with silver coated yarn (500 Ω/m)	hot melt and transfer molding of epoxy resin and hardener	no reduction in performance following 20 washes (ISO 6330:2000-6A: 40 °C, dripped dried); 19% failure outside encapsulated area
100% Nomex single jersey [80]	digital printed circuit board (10 Ω) soldered on electrodes of Kapton with Shieldtex (234/34-2 ply) silver coated polyamide (linear resistance < 100 Ωm)	silicon and thermoplastic polyurethane film	electrical conductivity decreased approximately four times following 50 wash cycles (ISO 6330—40 °C, 30 min)
99% polyester/1% carbon woven and polyamide/elastane knit [73]	standard packaged components and meander-shaped copper tracks covered with polyimide	four types of poly(dimethylsiloxane) (Dow Corning 9601, 9600, 184, 186 with different viscosities) applied by screen printing, cured 100 °C 10 min	washes as per ISO 6330:2000: Stable after 50 washes in protective bag, 60 °C water, 3 h (procedure 5A), water and soap (2.5 g/L standard detergent), gyro washing; functionality retained after five washes procedure 5A with protective bag, air dried; lost functionality after six washes (no protective bag, air dry); and two washes in protective bag with tumble drying; functional after five industrial washes at 40 °C with tumble drying 80 °C; functionality lost with 65 °C wash

## Supplementary Materials

The following are available online at https://www.mdpi.com/1424-8220/20/15/4243/s1, Figure S1: Appearance of water droplets over time (intervals selected to show change shape and absorption of water droplets) (**a**) Wool (**b**) Cotton, Figure S2: Effect of abrasion on the fabric surface (**a**) Wool, Table S1: Effects of functionalization on pill dimensions measured across to axes.
