# Supplementary material for: Encapsulation of Electrically Conductive Apparel Fabrics: Effects on Performance
Source: Sensors (Basel). 2020 Jul 30;20(15):4243. doi: 10.3390/s20154243 (PMC7436089; doi:10.3390/s20154243)
Supplement: Supplementary file 1 [file sensors-20-04243-s001.pdf]

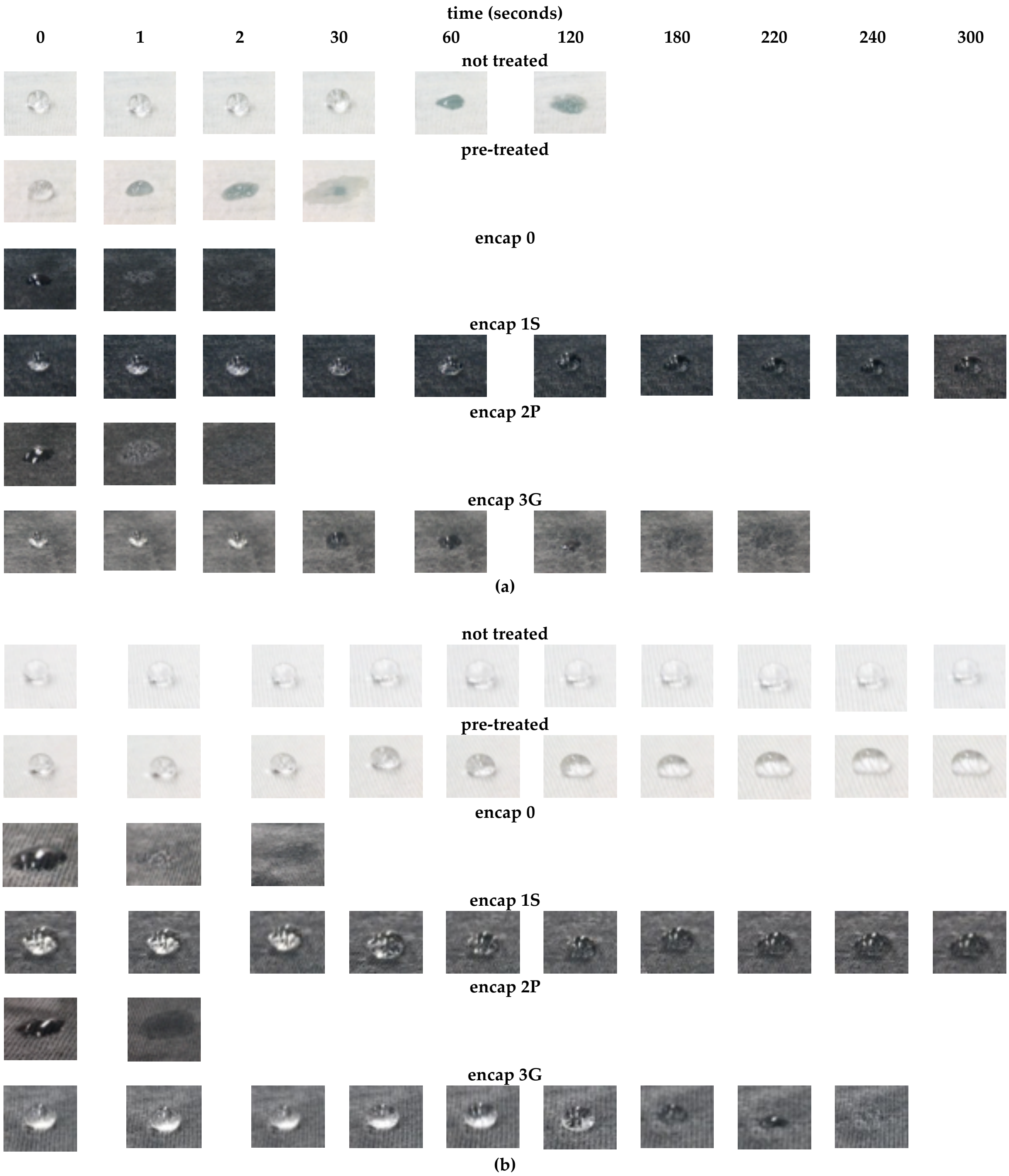

Figure S1. Appearance of water droplets over time (intervals selected to show change in shape and absorption of water droplets). **(a)** Wool. **(b)** Cotton.

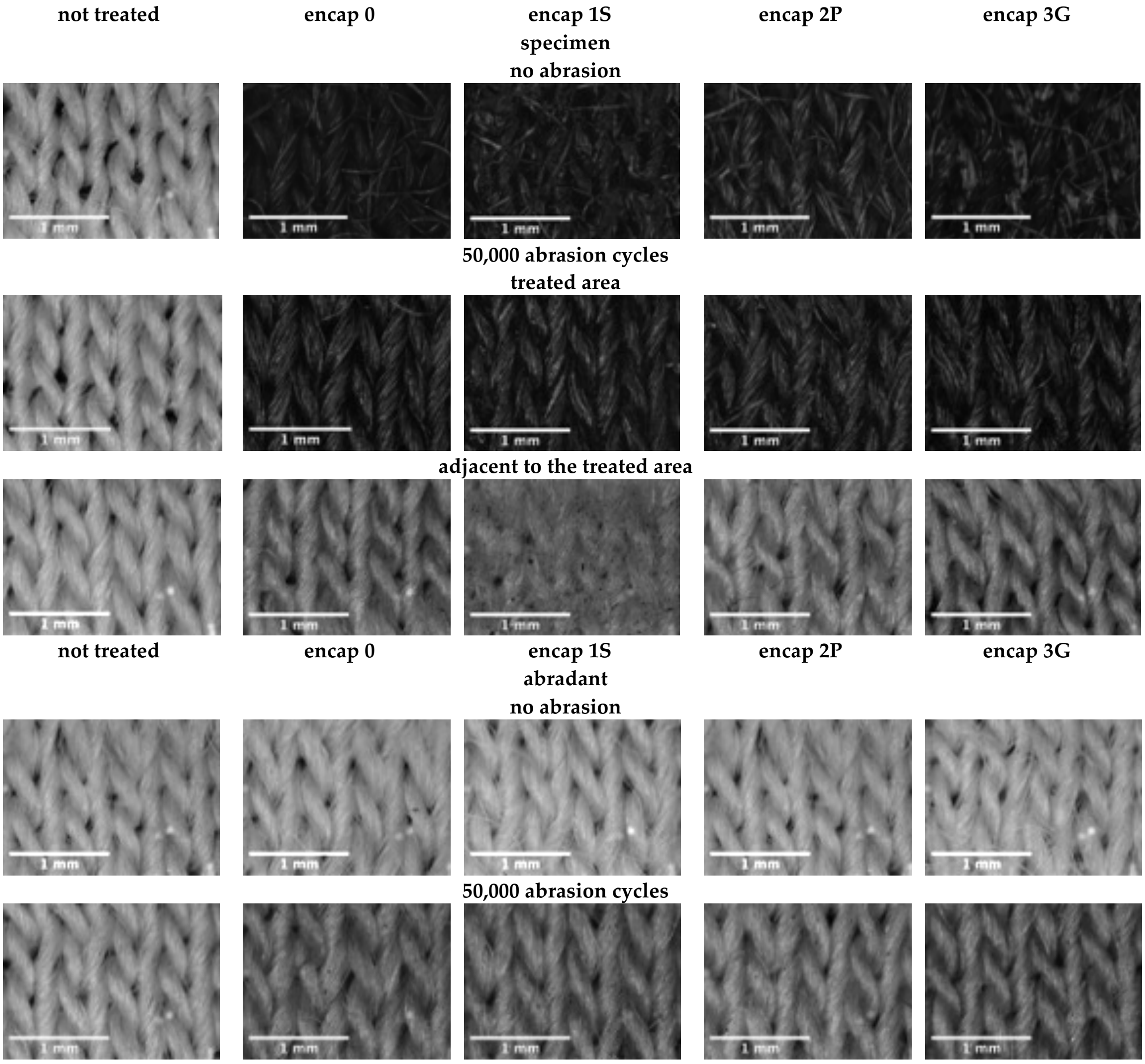

(a)

Figure S2. Effect of abrasion on wool fabric surface.
